# Clinical parameters and biomarkers predicting spontaneous operational tolerance after liver transplantation: a scoping review protocol

**DOI:** 10.12688/f1000research.21501.3

**Published:** 2020-06-26

**Authors:** Christian Appenzeller-Herzog, Steffen Hartleif, Julien Vionnet

**Affiliations:** 1 University Medical Library, University of Basel, Basel, 4051, Switzerland; 2 University Hospital for Children and Adolescents Tübingen, University Hospital Tubingen, Stuttgart, 70597, Germany; 3 Institute of Liver Studies, King's College Hospital, London, London, UK; 4 Transplantation Centre, University of Lausanne, Lausanne, Switzerland; 5 Service of Gastroenterology and Hepatology, University of Lausanne, Lausanne, Switzerland

**Keywords:** biomarker, clinical parameter, flow cytometry, gene expression profiling, immunosuppression, immunosuppression withdrawal, liver biopsy, liver transplantation, operational tolerance, regulatory lymphocytes, scoping review, Tregs

## Abstract

**Objective: **This scoping review aims at systematically mapping reported prognostic factors for spontaneous immunosuppression (IS) free allograft tolerance (operational tolerance, OT) in non-viral hepatitis and non-autoimmune disease liver transplant (LT) recipients who are undergoing immunosuppression withdrawal (ISW). The results may inform the subsequent conduct of a systematic review with a more specific review question.

**Background:** LT is currently the most effective treatment for end-stage liver diseases. Whereas the short-term outcomes after LT have dramatically improved over the last decades, the long-term outcomes remain unsatisfactory, mainly because of side effects of lifelong IS, such as infections, cardiovascular diseases, malignancies, and nephrotoxicity. ISW studies have shown that OT can be achieved by a subset of LT recipients and recent research has identified biomarkers of OT in these patients. However, an evidence-based selection algorithm for patients that can predictably benefit from ISW is not available to date. The planned review will, therefore, map existing knowledge on prognostic clinical parameters and biomarkers for OT.

**Inclusion criteria: **We will consider studies that record any clinical parameter or biomarker before the initiation of ISW in paediatric or adult non-viral hepatitis and non-autoimmune disease LT recipients and analyse their possible association with ISW outcomes (OT or non-tolerance). Studies addressing the effectiveness of OT-inducing treatments will be excluded.

**Methods:** Embase, MEDLINE, and Cochrane Library will be searched for relevant articles or conference abstracts. Full-texts of selected abstracts will be independently screened for inclusion by two reviewers. References and citing articles of included records will be screened for additional relevant records. Clinical trial registries will be searched for ongoing studies, and their investigators contacted for the sharing of unpublished data. Data from included records will be independently extracted by two reviewers using a prespecified data extraction table and presented in both tabular and narrative form.

## Introduction

Liver transplantation (LT) currently remains the only long-term treatment option for patients with end-stage liver failure. The success of LT was enabled by the introduction of effective pharmacological immunosuppressive strategies, which mostly target recipient T lymphocyte responses. The drugs that mediate immunosuppression (IS) in LT recipients exert their effects either by inhibiting intracellular T lymphocyte signalling or cellular proliferation. Calcineurin inhibitors (CNIs) ^1^ and mammalian target of rapamycin inhibitors (mTOR-I) ^2^ target the former, whereas corticosteroids ^3^ or antimetabolites like mycophenolate mofetil ^4^ or azathioprine ^5^ impair the latter. Moreover, biologic agents blocking the anti-interleukin 2 receptor on activated T lymphocytes (e.g., basiliximab ^6^) or inhibiting T lymphocyte costimulation (preliminary data in kidney transplantation: belatacept ^7^) have been developed more recently to reduce CNI exposure.

While providing effective protection against acute and chronic cellular rejection of the allograft, lifelong IS, particularly corticosteroids and CNIs, are known to cause significant side effects ^8^. Common side effects include various malignancies, cardiovascular and metabolic diseases, renal toxicity, as well as susceptibility to infections ^9– 14^. These significant side effects account for chronic morbidity and impair quality of life of LT recipients. Therefore, efforts to minimise exposure to immunosuppressive drugs while preserving graft integrity are warranted.

Among all solid organ transplants (SOT), the transplanted liver exhibits unique immunoregulatory properties, which render liver allografts less dependent on IS ^15– 17^. The attributed mechanisms of liver allograft tolerance are complex and may include deficient antigen presentation, large antigen load, neutralisation of alloantibodies, regulatory T cell (Treg) generation, and long-term microchimerism ^18– 22^. Accordingly, LT recipients usually require less intensive IS treatment with lower levels and/or numbers of immunosuppressive drugs compared to other SOT recipients ^23^. In addition, human leukocyte antigen (HLA) match requirements between donor and recipient are less stringent, and the incidence and severity of acute cellular rejection (ACR) episodes are lower and usually better tolerated in LT as compared to other SOT recipients ^24^.

Based on these particular features, clinical studies that examined IS minimization or even complete IS withdrawal (ISW) in LT recipients have been initiated already in the 1990s ^25– 28^. Most of these ISW studies (at least all of the recent ones) applied predefined eligibility criteria such as absence of recent rejection episodes or absence of significant histological lesions in a baseline biopsy ^29, 30^. In all studies, a significant subset of study participants exhibited stable allograft function and histological graft integrity despite complete ISW ^31^. In agreement with the nomenclature used in the literature, we herein call this state of spontaneous immunological transplant tolerance *operational tolerance* (OT) ^32^. However, the majority of study participants still would experience an ACR episode or develop abnormal liver function tests following ISW and eventually require the reinstitution of immunosuppressive drugs ^31^ (ISW failure). The mechanisms underlying ISW success or failure in LT recipients are currently not completely elucidated. Likewise, whether ISW outcomes may be predictable at all (see below) or IS minimisation is a safer alternative to complete ISW is not yet known ^33^.

The discovery of OT has promoted extensive research activity over the last two decades ^34^. On the one hand, it is important to explore the factors that are associated with or enable the development of OT in a subset of transplant recipients ^35^. More detailed knowledge on such predictors of spontaneous OT will help to refine the eligibility criteria for LT recipients to participate in ISW trials and hopefully increase the fraction of successful ISW attempts. On the other hand, researchers have started to address the question as to whether OT can be induced by immune manipulation prior to ISW. Thus, infusion of donor-derived hematopoietic stem cells ^36– 40^, Treg ^41^, regulatory dendritic cells (DCreg) ^42^ or mesenchymal stem cells ^43, 44^, as well as lymphodepletion protocols using T lymphocyte-directed antibodies ^45^ have been or are being tested for their potential to induce tolerance ^31^.

### Why it is important to do this review?

Regarding the therapeutic dilemma of deleterious effects of chronic IS vs. the risk of ISW failure and graft injury after LT, there is a medical need to define clinical and biochemical markers to predict the success of ISW. Up to now, there is only one systematic review that addressed the benefits and harms of ISW in LT recipients ^46^. It focused on CNI and included only randomized controlled trials (RCTs) comparing ISW and IS continuation after LT. The authors identified a single ongoing RCT, which has been published in the meantime ^47^. In this RCT, the non-inferiority analysis of ISW vs. unchanged IS maintenance treatment on a composite morbidity/mortality endpoint was inconclusive. Based on these results and an unpublished scoping search in the literature that did not identify any new RCTs on this comparison, we concluded that there was not enough data for a new systematic review approach comparing ISW and IS continuation after LT.

In contrast, the number of publications that highlight predisposing factors or biomarkers for spontaneous OT in ISW cohorts is increasing ^35^. We, therefore, reasoned that the systematic scoping for evidence on such factors would best inform the community regarding the therapeutic dilemma of IS after LT. Accordingly, this scoping review will for the first time systematically collect biomarkers and clinical parameters that are likely predictors of spontaneous OT. The anticipated results shall set the basis for subsequent evidence syntheses or clinical trials with a sharpened research focus. Any evidence that will help understand the spontaneous development of OT and increase the fraction of successful ISW by enabling an informed preselection of ISW candidates is of great value to the community, as it will provide valuable guidance in the therapeutic dilemma of IS after LT.

### Study aim and objectives/questions

The objective of this scoping review will be to map all published prognostic factors for spontaneous OT in non-viral hepatitis and non-autoimmune disease LT recipients who are undergoing ISW. The obtained results may inform the subsequent conduct of a systematic review with a more targeted review question.

Specifically, the review questions are:

i) What are clinical parameters and biomarkers that predispose LT recipient ISW candidates to achieve spontaneous OT?
ii) What are the success rates of ISW and achievement of spontaneous OT in LT recipients?
iii) What are the rates of graft loss in LT recipients following ISW?

## Protocol

### Data collection

***Eligibility criteria***

**Population, Intervention, Outcomes**

The primary eligibility criterion will be the assessment of spontaneous OT, i.e. rejection-free liver allograft survival for at least one year following ISW. LT recipients of any age or stage will be included, but recipients with underlying autoimmune diseases, replicative viral disease and/or multi-organ recipients will be excluded. Studies reporting on mixed populations will be included, if less than 20% of the study population has a replicative viral liver disease or an autoimmune liver disease aetiology. Studies that do not report the liver disease aetiologies for LT in their population will also be included. All pharmacological IS regimens including combination treatments being completely withdrawn will be eligible. However, studies addressing dose reduction of IS including IS minimisation, withdrawal of a subset of drugs from IS combination treatments (e.g. withdrawal of corticosteroids in patients on CNI maintenance treatment), or conversion between IS regimens (e.g. CNI to mTOR-I conversion vs. CNI continuation) will be excluded.

We will include studies that assess an association of pre-ISW clinical parameters or biomarkers on the development of OT. Studies exclusively addressing the effectiveness of induction or immunomodulation therapies for development of OT (using lymphodepletion or infusion with regulatory immune cells) will be excluded. Prespecified pre-ISW clinical parameters potentially predicting OT are sex, recipient age at LT, time since LT, history of episodes of rejection, liver histology, pharmacologic IS regimen, living (LD) or deceased donor (DD) LT, degree of kinship (or HLA match) of the donor, lymphocytotoxic crossmatch, liver disease aetiology, and previous pregnancies, SOT, or blood transfusions. Prespecified pre-ISW biomarkers potentially predicting OT are any up- or downregulated immune cell subsets (detected by flow cytometry or mass cytometry), any up- or downregulated genes or micro RNAs in the liver allograft or peripheral blood (detected by gene microarray, quantitative PCR, or RNA-seq), epigenetic markers, and anti-HLA antibodies (detected by ELISA, single antigen bead assay, or complement-dependent cytotoxicity assay). Owing to the risk of confounding by interrupted IS in the OT cohort (i.e. featuring successful ISW), data on post-ISW biomarkers will be excluded unless the same biomarkers were measured in the same patients already before ISW.

**Types of study to be included**

We will include prospective, retrospective, randomised, and non-randomised studies irrespective of publication status and including case-control and cross-sectional designs. By reporting on those patients that did not achieve OT after ISW most relevant studies would include a “control cohort” by default. Principal investigators of ongoing studies and conference abstracts will be contacted twice by email for the sharing of any unpublished data. If no additional information can be obtained, these records will be tabulated along with a disclaimer notice. Conference abstracts and trial registry entries where the data was subsequently published in a peer-reviewed article will be excluded. Animal studies, case reports, case series (i.e. publications where patient histories of exclusively tolerant or non-tolerant ISW-liver recipients are reported ^48^), reviews, letters, and editorials will be excluded. No language or publication date restrictions will be applied.

***Identification of relevant literature.*** An information specialist (CA-H) will develop the search strategies, which will be reviewed by a second information specialist. Database-specific subject headings and text words (synonyms and word variations) for liver transplantation, ISW, and OT, graft survival, or liver biopsy will be used. We will search the electronic databases Embase via Elsevier, Medline via Ovid, and the Cochrane Central Register of Controlled Trials (CENTRAL). The search string for Embase is provided in Box 1. We will also search the study registry clinicaltrials.gov as well as the World Health Organization’s International Clinical Trials Registry Platform (ICTRP) for ongoing studies. All retrieved references will be exported to EndNote X9 and deduplicated.

Box 1. **Search strategy for Embase**

(‘liver transplantation’/exp OR (OLT OR LTx):ab,ti OR ((‘liver’/de OR ‘liver lobe’/exp OR ‘liver disease’/exp OR ‘obstructive bile duct disease’/exp OR ‘bile duct atresia’/de OR (liver OR hepatic OR hepato* OR hepatis OR hepatitis OR intrahepatic OR extrahepatic OR cirrhosis OR cirrhotic OR ‘periportal fibrosis’ OR jaundice OR icterus OR bilirubinaemia OR cholestasis OR cholestatic OR ((bile OR biliary OR choledoch*) NEAR/3 (obstruction OR stasis OR occlusion OR stenosis OR stricture OR obliteration OR atresia OR agenesi*))):ab,ti) AND (‘transplantation’/de OR ‘organ transplantation’/de OR ‘allotransplantation’/de OR ‘orthotopic transplantation’/de OR ‘recipient’/exp OR (transplant* OR Tx OR allotransplant* OR graft* OR allograft* OR recipient*):ab,ti)))

AND

(((‘immunosuppressive agent’/exp OR ‘calcineurin inhibitor’/exp OR ‘mammalian target of rapamycin inhibitor’/de OR ‘immunosuppressive treatment’/de) AND (‘treatment withdrawal’/exp OR ‘weaning’/de)) OR ((((immunosuppress* OR immuno-suppress* OR immune-suppress* OR immunodepress* OR immuno-depress* OR immune-depress* OR anti-rejection OR antirejection OR ‘immune system-suppressing’ OR ‘transplantation reaction inhibition’ OR anti-metaboli* OR antimetaboli* OR azathioprine OR belatacept OR cyclophosphamide OR daclizumab OR ‘mycophenolate mofetil’ OR MMF OR ‘mycophenolic acid’ OR cellcept OR ‘calcineurin inhibitor*’ OR ‘protein phosphatase 2B inhibitor*’ OR cyclosporin* OR ciclosporin* OR neoral OR sandim* OR tacrolimus OR advagraf OR prograf* OR fk506 OR fk-506 OR ‘mammalian target of rapamycin inhibitor*’ OR ‘mammalian target of rapamycin kinase inhibitor*’ OR ‘mechanistic target of rapamycin inhibitor*’ OR ‘mechanistic target of rapamycin kinase inhibitor*’ OR ‘mTOR inhibitor*’ OR ‘mTOR kinase inhibitor*’ OR everolimus OR rad001* OR rad-001* OR rapamune OR rapamycin OR sirolimus) NEAR/4 (withdraw* OR taper* OR wean* OR minimization OR minimisation OR minimizing OR minimising OR sparing OR eliminat* OR reduction OR reducing OR lower* OR cessation OR discontinu* OR interrupt* OR abstinence OR avoid* OR stop* OR downgrad* OR diminish* OR free*)) OR is-withdraw* OR is-taper* OR is-wean* OR is-minimization OR is-minimisation OR is-minimizing OR is-minimising OR is-sparing OR is-eliminat* OR is-reduction OR is-reducing OR is-lower* OR is-cessation OR is-discontinu* OR is-interrupt* OR is-abstinence OR is-avoid* OR is-stop* OR is-downgrad* OR is-diminish* OR is-free):ab,ti))

AND

(‘transplantation tolerance’/de OR ‘immunological tolerance’/de OR ‘immunoregulation’/de OR ‘immunoreactivity’/de OR ‘graft survival’/de OR ‘liver biopsy’/de OR (tolerogen* OR ‘tolerant patient*’ OR ‘tolerant state’ OR ‘state of tolerance’ OR ‘sustained weaning’ OR ((transplant* OR posttransplant* OR operational* OR immune OR immunologic* OR alloimmune OR allograft* OR graft* OR alloantigen* OR antigen* OR chimerism OR donor-specific OR peripheral) NEAR/3 (tolerance OR tolerant OR tolerated OR tolerating OR acceptance OR protect* OR quiescen* OR unresponsive* OR nonresponsive* OR un-responsive* OR non-responsive*)) OR immunoregulat* OR immunosurveill* OR immunoreactiv* OR immunoactiv* OR ((immune OR immunologic*) NEXT (regulat* OR surveill* OR reactiv* OR activ*)) OR ((graft OR allograft OR transplant* OR liver OR hepatic) NEAR/3 (survival OR health OR function OR ‘resistance to rejection’)) OR ((inhibit* OR decrease OR abolish OR suppress* OR reduc* OR ameliorat* OR improve* OR absent OR avoid* OR prevent*) NEAR/3 (graft OR allograft OR transplant* OR liver OR hepatic) NEAR/3 (injury OR complication* OR dysfunction OR inflammation OR fibrosis OR infiltration)) OR ((inhibit* OR decrease OR abolish OR suppress* OR reduc* OR ameliorat* OR improve* OR absent OR avoid* OR prevent*) NEAR/3 (rejection OR ‘immune response*’ OR ‘alloimmune response*’ OR ‘T-cell response*’ OR ‘B-cell response*’ OR ‘antibody response*’ OR ‘humoral response*’)) OR ((liver OR hepatic) NEAR/3 (biopsy OR biopsies OR puncture*))):ab,ti)

NOT

((‘animal’/de OR ‘animal experiment’/exp OR ‘nonhuman’/de) NOT (‘human’/exp OR ‘human experiment’/de))

**NOTE: The subject heading “graft rejection” (and respective free text terms) was omitted from the third search block, because its tentative inclusion resulted in a non-manageable increase of hits.**

One reviewer (CA-H) will screen the deduplicated references based on their titles and abstracts. All potentially relevant references will be retrieved in full-text and independently assessed by two reviewers (CA-H, JV). Any disagreements over eligibility will be resolved by consensus. Where necessary, a third review author (SH) will make a final judgement. All judgements at the full-text screening stage will be collected in a standardised MS Excel 2016 form. Articles in foreign languages that none of the review authors is familiar with will be checked for eligibility by other researchers before translation will be considered. 

To identify possible additional studies that will escape our electronic database searches, we will screen the bibliographic references and the citations of all included articles that are indexed in Scopus or the Web of Science.

### Data analysis

***Quality appraisal.*** Within the framework of this scoping review, no quality appraisal is planned.

***Data charting.*** Next to reported prognostic and non-prognostic factors (clinical parameters and biomarkers) for OT, which will be the primary outcomes, we will also chart the percentage of successful ISW and achievement of sustained OT and the rate of graft loss in each trial as the secondary outcomes. Two reviewers (CA-H, JV) will independently chart the data from each eligible article using a jointly developed MS Excel 2016 charting form that will be pilot tested using four eligible full-text articles. If necessary, the charting form will be updated in an iterative process. Any disagreements will be solved by discussion. Data will be sought for the following variables:

- Article characteristics such as first author, year of publication, country of origin, and bibliographic details
- Funder
- Trial ID
- Mono- or multicenter study
- Study design, IS maintenance control group yes/no
- Study population paediatric/adult/mixed
- DD and/or LD LT
- Recipient age at LT
- Donor age
- Liver disease aetiology
- Viral status during ISW
- Time from LT to ISW
- Duration of follow-up
- IS drug(s)
- Reason(s) for ISW elective/non-elective
- ISW schedule
- Method(s) for assessing OT
- Total number of patients that are included in the prognostic analyses
- Percentage of successful ISW (achievement of OT)
- Percentage of graft loss
- Biomarkers predicting OT
- Clinical parameters predicting OT
- Numerical evidence for positive associations
- Biomarkers explicitly not predicting OT
- Clinical parameters explicitly not predicting OT

***Strategy for data synthesis and presentation.*** For each included article, ongoing study, or conference abstract with data, we will present the charted data in a “results of individual sources of evidence” table. For the synthesis of collated prognostic factors (biomarkers and clinical parameters) for OT, we will use descriptive statistics showing the individual sources of evidence that support each factor. In addition to a tabular view, the results will be narratively synthesized in the review text. Together, these results will provide a comprehensive scope of past research activity on this topic and likely identify promising future research avenues.

### Design and reporting guidelines

This scoping review will be conducted along with the guidelines by the Joanna Briggs Institute ^49^ and reported according to the “Preferred Reporting Items for Systematic reviews and Meta-Analyses extension for Scoping Reviews” (PRISMA-ScR) statement ^50^.

### Dissemination of results

The completed review will be published in a peer-reviewed journal.

## Study status

Start date of search: July 2019; anticipated completion date of review: September 2020.

Current study status: preliminary searches, yes; piloting of the study selection process, yes; formal screening of search results against eligibility criteria, started; data extraction, no; data analysis, no.

## Conclusions

Since the first reports of spontaneous OT in LT ^25– 28^, numerous studies of ISW have been published (reviewed in 32). These studies were initially uncontrolled and heterogeneous in their design, rendering any comparison between them and any conclusions difficult to draw. Following the creation of international consortia (Immune Tolerance Network – ITN – in the US and Reprogramming the Immune System for the Establishment of Tolerance – RISET – in Europe), inclusion/exclusion criteria of ISW studies in LT have been harmonised, thus allowing cross-comparisons and cross-validations between studies. For instance, two ongoing multicenter trials (LIFT ^51^ and OPTIMAL ^52^) share the same inclusion/exclusion criteria.

These ISW trials have in parallel fuelled the need to find reliable biomarkers for the identification of those patients who are more likely to successfully stop IS, a problem that is most critical for the safety and future applicability of ISW. While clinically not available yet, several biomarkers have already been evaluated in LT recipients. In this scoping review, we will map all information on this body of literature. In addition to that, our searches in clinical trial registries will provide an overview of the current research activity in the field. The anticipated results will allow us to determine possible research gaps and whether any future systematic reviewing and meta-analysis efforts are warranted.

## Data availability

### Underlying data

No data are associated with this article.
