# Supplementary material for: Clinical parameters and biomarkers predicting spontaneous operational tolerance after liver transplantation: a scoping review protocol
Source: F1000Res. 2020 Jun 26;8:2059. Originally published 2019 Dec 5. [Version 3] doi: 10.12688/f1000research.21501.3 (PMC7194345; doi:10.12688/f1000research.21501.3)
Supplement: Peer review history

# Peer review: Reviewer response for version 2

**DOI:** 10.5256/f1000research.24506.r61649

**Published:** 2020-04-22

**Authors:** Piotr Czubkowski

**Affiliations:** 1 Department of Gastroenterology, Hepatology, Feeding Disorders and Pediatrics, The Children’s Memorial Health Institute, Warsaw, Poland

The Authors performed the scoping review clarifying the ways of obtaining and analyzing data on operational tolerance in liver transplant recipients, both adults and children. The aim of this comprehensive review is to identify prognostic factors, clinical parameters and biomarkers, for spontaneous operational tolerance after complete immunosuppression withdrawal. This is extremely interesting topic from scientific point of view but even more then that it has high relevance from patient’s perspective, especially children for whom lifetime immunosuppression is significant burden.

 Nevertheless, this task requires high quality data, clear inclusion criteria and cautious interpretation. 

1. Do the Authors plan to pool adult and pediatric data together or review it separately? Interpolation of adult data for children should be avoided.
2. Eliminating autoimmune or viral etiologies may cause selection bias – what was the rationale for this criterion? If manuscripts with unknown etiology or less then 20% of autoimmune/viral are eligible for the study, this criterion should be more clarified or maybe just simplified.
3. I’d be cautious in adding conference abstracts, or maybe the Authors should exclude abstracts older then 2-3 years which were not published so far or direct contact with the Authors of conference abstracts should be considered for data clarification and update. 

In terms of study aims/objectives:

1. Success rate of ISW: how the Authors plan to define the outcomes, regarded different efficacy endpoints among the studies (minimum period of follow-up without rejection, protocol liver biopsy)?

Is the study design appropriate for the research question?

Partly

Is the rationale for, and objectives of, the study clearly described?

Yes

Are sufficient details of the methods provided to allow replication by others?

Yes

Are the datasets clearly presented in a useable and accessible format?

Not applicable

Reviewer Expertise:

Pediatric hepatology and liver transplantation

I confirm that I have read this submission and believe that I have an appropriate level of expertise to confirm that it is of an acceptable scientific standard.

# Response

**Published:** 2020-06-23

**Authors:** Christian Appenzeller-Herzog

**Affiliations:** University of Basel, Switzerland

Dear Prof. Czubkowski, dear Piotr,

We are grateful for the helpful reviewer report you provided and, in particular, for your valid suggestions for improvement of our scoping review protocol. We have used these suggestions as a basis to create version3 of the protocol. The changes that we implemented in version 3 in response to your comments are detailed below in a point-by-point response:

Comment:

    Do the Authors plan to pool adult and pediatric data together or review it separately? Interpolation of adult data for children should be avoided.

*Answer:*

*No, we do not plan to pool adult and paediatric data. In fact, we believe that the planned juxtaposition of collected OT-predicting clinical parameters and biomarkers in children and adults may be a strength of the scoping review. In response to your important comment, we have added the charting item “Study population paediatric/adult/mixed” to our list for data charting. This will facilitate the categorization of identified prognostic factors into age groups and may visualize possible differences (or highlight the need for additional research).*

*(Besides, we have also added the charting item “Reason(s) for ISW elective/non-elective”.)*

Comment:

    Eliminating autoimmune or viral etiologies may cause selection bias – what was the rationale for this criterion? If manuscripts with unknown etiology or less then 20% of autoimmune/viral are eligible for the study, this criterion should be more clarified or maybe just simplified.

*Answer:*

*Systematic exclusion of autoimmune liver aetiologies is well acknowledged in the field of operational tolerance (OT) in liver transplantation because of the propensity of this population to develop rejection when immunosuppression withdrawal is attempted. In other words, the prevalence of OT is tremendously low in this population. The reason for exclusion of patients with active viral hepatitis is the specific reprogramming of the immune system that takes place in these patients. This reprogramming that is manifest e.g. by overt transcriptional changes (see Bohne et al., JCI 2012 for viral hepatitis patients) will certainly have an impact on the composition and validity of OT-predicting biomarkers. Thus, we feel that aiming at a homogeneous population without such background will increase the validity of our final results for non-autoimmune/non-viral liver recipients rather than cause selection bias. Furthermore, these autoimmune/viral exclusion criteria are in agreement with the criteria applied by current ISW trials like e.g. the LIFT trial.*

*The published evidence of OT-predicting factors is expected to be rather scarce, and we must make sure that our pre-specified exclusion criteria will not eliminate any key studies in the field (although they might not perfectly fit our scope). For example, the Bohne-study cited above included 6% and 15% patients with HCV infection in the non-tolerant and tolerant cohort, respectively. At the same time, this study is probably one of the richest reservoirs for the extraction of OT-predicting biomarkers. On the basis of this, we formulated our exclusion criteria with a 20% cut-off and also with the decision to include patients with non-reported liver disease aetiology. These “soft” exclusion criteria will be acknowledged as limitations of our study in the final report.*

*To make our exclusion criteria referring to this issue clearer (especially concerning active replicative viral disease) we have reformulated the respective sentence, which now reads:*

*“Studies reporting on mixed populations will be included, if less than 20% of the study population has a replicative viral liver disease or an autoimmune liver disease aetiology.”*

*In addition, we have added the charting item “Viral status during ISW” to our list for data charting.*

Comment:

    I’d be cautious in adding conference abstracts, or maybe the Authors should exclude abstracts older then 2-3 years which were not published so far or direct contact with the Authors of conference abstracts should be considered for data clarification and update.

*Answer:*

*Indeed, we are planning to contact authors of conference abstracts by email. The sentences referring to this have been refined and slightly extended:*

*“Principal investigators of ongoing studies and conference abstracts will be contacted twice by email for the sharing of any unpublished data. If no additional information can be obtained, these records will be tabulated along with a disclaimer notice. Conference abstracts and trial registry entries where the data was subsequently published in a peer-reviewed article will be excluded.”*

Comment:

    Success rate of ISW: how the Authors plan to define the outcomes, regarded different efficacy endpoints among the studies (minimum period of follow-up without rejection, protocol liver biopsy)?

*Answer:*

*In our review, we define OT as “rejection-free liver allograft survival for at least one year following ISW”. But the reviewer is right that this definition is not uniformly adopted in all published ISW studies. Non-uniform definitions of OT will be formulated as a limitation of our review. However, since no quantitative meta-analysis is planned, we feel that a firm plan on how to deal with other outcome definitions is not required.*

Thank you again for your critical feedback in the reviewer report. We hope that you agree with us and that the changes we made in version 3 fully meet your concerns.

Christian Appenzeller-Herzog, Steffen Hartleif, Julien Vionnet

# Peer review: Reviewer response for version 2

**DOI:** 10.5256/f1000research.24506.r59244

**Published:** 2020-02-07

**Authors:** Deirdre Kelly

**Affiliations:** 1 Liver Unit, Birmingham Children's Hospital NHS Foundation Trust, Birmingham, UK

Is the study design appropriate for the research question?

No

Is the rationale for, and objectives of, the study clearly described?

Yes

Are sufficient details of the methods provided to allow replication by others?

Yes

Are the datasets clearly presented in a useable and accessible format?

Not applicable

Reviewer Expertise:

NA

I confirm that I have read this submission and believe that I have an appropriate level of expertise to confirm that it is of an acceptable scientific standard.

# Peer review: Reviewer response for version 1

**DOI:** 10.5256/f1000research.23689.r57506

**Published:** 2020-01-13

**Authors:** Deirdre Kelly

**Affiliations:** 1 Liver Unit, Birmingham Children's Hospital NHS Foundation Trust, Birmingham, UK

This scoping review systematically sets out how the Authors will consider studies that will record clinical details or biomarkers of relevance in the initiation of immunosuppression withdrawal.

 It is an ambitious review and although the Authors have laid out their strategy quite clearly by searching the relevant registries, databases and publications they do not specify whether they will include studies in children as well as adults. Most of the references are adult studies, so presumably this is the focus.

 I think the Authors will need to differentiate between IS minimisation or complete IS withdrawal and be clear which definitions they will include in their review for analysis..

 With regard to the review questions:

- The clinical parameters and biomarkers (Question 1)  may be difficult to clearly identify whereas review questions 2 and 3 are more likely to be clearly defined in studies and databases.

The relevance of this review and its potential benefit to patients is obvious, I am concerned that it will require an extensive amount of work without identifying a clear outcome of value to Clinicians or Patients.

Is the study design appropriate for the research question?

No

Is the rationale for, and objectives of, the study clearly described?

Yes

Are sufficient details of the methods provided to allow replication by others?

Yes

Are the datasets clearly presented in a useable and accessible format?

Not applicable

Reviewer Expertise:

NA

I confirm that I have read this submission and believe that I have an appropriate level of expertise to confirm that it is of an acceptable scientific standard, however I have significant reservations, as outlined above.

# Response

**Published:** 2020-01-27

**Authors:** Christian Appenzeller-Herzog

**Affiliations:** University of Basel, Switzerland

Dear Prof. Kelly,

Thank you very much for your constructive reviewer report and for drawing our attention to different issues in our protocol and possibilities for improvement. We are most happy with your judgements that this ambitious review has an obvious potential benefit to patients and that our protocol paper is of an acceptable scientific standard. With regard to your criticism, we would like to address the three points you raised: i) pediatric or adult patients?, ii) IS withdrawal and minimisation?, iii) difficulties to cope with review question 1:

- Pediatric/adult: This review will include all ISW studies irrespective of LT recipient age. Our protocol already states in the subsection “population, intervention, outcomes” that “LT recipients of any age or stage will be included”. To make this clearer to the reader we have now also added this information in the abstract, which now reads: “We will consider studies that record any clinical parameter or biomarker before the initiation of ISW in paediatric or adult non-viral hepatitis and non-autoimmune disease LT recipients …”

The question whether or not OT-predicting factors differ in children and adults is interesting and important. Potentially, the planned systematic mapping of published predicting factors could provide some clues on this in the final review.

- IS withdrawal/minimisation: This review will only focus on full ISW studies and exclude studies on IS minimisation. Our protocol already holds the following statement in the subsection “population, intervention, outcomes”: “However, studies addressing dose reduction of IS, (…) will be excluded”. To clarify that also IS minimization studies will be considered ineligible we have now implemented the following extension: “However, studies addressing dose reduction of IS including IS minimisation, (…) will be excluded”.
- Review question 1: This is an important issue and we thank you for pointing this out. The objective of this scoping review is to collect and map all available evidence of OT-predicting factors. If no such factors can be identified, this result, albeit negative, will still be of importance and worth reporting. However, as you are pointing out, the formulations in our protocol are implying that our aim is to “identify” such factors in the literature. Instead, we should state that our objective is the mapping of all available evidence on such factors. In response to this important point of criticism, we have implemented the following revisions: Abstract: “This scoping review aims at systematically mapping reported prognostic factors for spontaneous immunosuppression (IS) free allograft tolerance (operational tolerance, OT) in non-viral hepatitis and non-autoimmune disease liver transplant (LT) recipients who are undergoing immunosuppression withdrawal (ISW).” Study aim and objectives/questions: “The objective of this scoping review will be to map all published prognostic factors for spontaneous OT in non-viral hepatitis and non-autoimmune disease LT recipients who are undergoing ISW.”

We sincerely hope that these amendments to our protocol will help convince you to fully approve the revised version of our manuscript. Thank you again for your invaluable help in this matter.

Christian Appenzeller-Herzog, Steffen Hartleif, Julien Vionnet
